# Organ-specific pre-metastatic and metastatic niches in colorectal cancer: “discrepancy in response to immune checkpoint inhibitors in liver and lung metastasis”

**DOI:** 10.3389/fimmu.2026.1838169

**Published:** 2026-05-21

**Authors:** Mohamad Mourad, Layal Al Mahmasani, Noura Abbas, Ali Shamseddine

**Affiliations:** Department of Internal Medicine, Hematology/Oncology Division, American University of Beirut Medical Center, Beirut, Lebanon

**Keywords:** colorectal cancer, immune checkpoint inhibitors, liver metastasis, lung metastasis, pre-metastatic niche, tumor microenvironment

## Abstract

Colorectal cancer (CRC) remains a leading cause of cancer-related mortality worldwide, largely driven by metastatic disease. Increasing evidence suggests that metastasis is dictated by tumor-secreted factors that shape the microenvironment of target organs leading to the formation of the pre-metastatic niche (PMN) and resultant metastatic niche and tumor microenvironment (TME). Tumor-derived factors, including extracellular vesicles, cytokines, and integrins, orchestrate vascular remodeling, immune suppression, and stromal reprogramming in distant organs prior to tumor cell arrival. While many of these processes are shared, the liver develops a highly tolerogenic and immunosuppressive niche characterized by myeloid cell accumulation, T-cell depletion, and systemic immune dampening. In contrast, the lung exhibits a more inflammatory and immune-reactive microenvironment, with enhanced lymphocyte activation but concurrent neutrophil-driven immunosuppression. These differences translate into clinically meaningful disparities in immunotherapy outcomes, with liver metastasis consistently associated with resistance to ICIs, whereas lung metastasis demonstrates relatively improved responses. This review examines the mechanisms underlying PMN formation in CRC, with a particular focus on the liver and lung, and explores how their distinct immune landscapes influence response to immune checkpoint inhibitors (ICIs). It also evaluates the current therapeutic strategies aimed at overcoming organ-specific resistance, including combination immunotherapies, anti-angiogenic agents, targeted therapies, and TME-modulating approaches. Finally, we highlight key limitations in existing evidence and propose future directions, emphasizing the need for organ-specific therapeutic strategies and clinical trial designs. A deeper understanding of PMN biology and organ-dependent immune regulation may enable more effective, tailored treatments for metastatic CRC.

## 1 Introduction

Globally, colorectal cancer (CRC) ranks third in incidence, with 1.9 million new cases annually, and is the second leading cause of cancer-related mortality with almost 900,000 deaths yearly (1). Around 15% to 30% of patients are diagnosed with metastatic disease at presentation, while 20%–50% of those with initially localized tumors eventually develop metastasis. The liver is the most common metastatic site, followed by the lungs, peritoneum, and distant lymph nodes (2).

Since metastasis is the major cause of cancer-related mortality, the mechanisms of metastasis have been investigated for decades. The “seed and soil” theory was first suggested by Paget in 1889, in which dissemination of tumor cells (seed) leads to metastasis only when the distant organ microenvironment (soil) is favorable (3). To achieve this, tumor cells precondition distant organs and induce immunosuppression and stromal remodeling, thereby forming what is known as the pre-metastatic niche (PMN). Ultimately, the cellular and molecular interactions shape the tumor microenvironment (TME) and influence tumor progression, metastatic spread and treatment response (4). Metastatic progression is therefore not solely determined by tumor-intrinsic genetic alterations but is strongly influenced by the immune and stromal composition of the metastatic organ. The immune microenvironment varies substantially between anatomical sites, shaping whether disseminated tumor cells are eliminated, remain dormant, or progress into overt metastasis (5). These organ-specific immune niches consist of distinct populations of immune cells, cytokine signaling pathways, and stromal components that can either promote or suppress tumor growth. As a result, the biological behavior of metastatic tumors and their responsiveness to therapy may differ depending on the organ in which they reside (6).

In CRC, response to immune checkpoint inhibitors (ICIs) is primarily determined by multiple factors. The microsatellite instability status is a molecular alteration that has a major influence on immune responsiveness. Tumors that exhibit high microsatellite instability (MSI-H)/deficient mismatch repair (dMMR) benefit the most from ICIs, compared to microsatellite stable (MSS)/proficient mismatch repair (pMMR) tumors (7, 8). Several additional features correlate with improved immunotherapy responsiveness such as increased number of tumor-infiltrating lymphocytes, high Immunoscore, active interferon-gamma signaling, high tumor mutational burden, and POLE mutations (9, 10). Within each of the molecular and genetic subtypes of CRC, the anatomical site of metastatic disease is increasingly recognized as a key determinant of ICI response. This variability may depend on the presence or absence of metastases in specific organs, potentially reflecting differences that arise during PMN formation (11).

Tumor cells secrete a variety of mediators that induce changes in the microenvironment of potential distant metastatic sites to become favorable for the engraftment of circulating tumor cells (12). Since immune landscapes differ among different organs, the PMN and the resultant TME within them also differ. For example, the immune-tolerant microenvironment of the liver prevents excessive inflammation from gut-derived antigens and microbes, whereas the lungs maintain robust immune surveillance to respond to inhaled pathogens (13). Indeed emerging evidence in CRC suggests that the anatomical site of metastasis may influence response to ICIs, where lung metastasis frequently demonstrates more favorable responses to ICIs compared to liver metastasis, highlighting the potential role of organ-specific immune environments in determining treatment outcomes (14). This observation raises the possibility that differences in the immune architecture of the pre-metastatic and metastatic niches between organs may contribute to heterogeneous responses to immunotherapy in metastatic CRC. Understanding how tumor-derived signals shape the immune microenvironment of different organs may therefore provide critical insights into mechanisms of resistance to ICIs and guide the development of more effective therapeutic strategies. Given the central role of the tumor microenvironment in metastatic progression and immune escape, ICIs represent a critical therapeutic strategy, with growing evidence that their efficacy is influenced by organ-specific differences in metastatic immune niches. The aim of this review is to explore the mechanisms of formation of PMN of CRC and the resultant TME in the liver and lung, and how these differences explain the discrepancy in ICI response between these organs.

## 2 Common pathway of PMN formation

The PMN formation is the process by which the primary tumor conditions distant organs to receive metastatic cells. In CRC, the process begins with the release of tumor-derived factors, including VEGF-A, TGF-β, TNF-α, interleukins, and S100 family proteins, as well as extracellular vesicles (EVs) including exosomes, RNAs, and other signaling molecules that reach distant tissues via the circulation (15). These factors affect organ vasculature and upregulate endothelial adhesion molecules, promoting angiogenesis and establishing an early permissive microenvironment (16).

Once secreted by the tumor cells, EVs are internalized by endothelial cells in pre-metastatic organs leading to an increase in vascular permeability and upregulation of adhesion molecules, thus facilitating circulating tumor cell extravasation and engraftment (17). These signals also drive extracellular matrix (ECM) remodeling, by enzymes such as lysyl oxidase, which crosslinks collagen and enhances myeloid cell adhesion, creating a scaffold for future tumor cells that is characterized by increased stiffness, fibronectin deposition, and integrin-mediated signaling (18). Some mediators within these exosomes are organ-specific: integrin signatures such as α6β4 and α6β1 have been associated with lung tropism, and αvβ5 indicate liver tropism (19). Interestingly, pre-clinical studies have demonstrated that modifying exosome integrin composition can redirect metastasis to alternate organs, highlighting their functional role in organ-specific PMN formation (19).

A hallmark of early PMN formation is the recruitment of bone marrow-derived cells. VEGFR1^+^ hematopoietic progenitor cells accumulate in future metastatic sites before tumor cell arrival, creating adhesive, chemoattractive microenvironments that facilitate later seeding (20). Myeloid-derived suppressor cells (MDSCs) are also recruited, where they exert immunosuppressive effects that inhibit adaptive antitumor immunity and foster immune tolerance within the niche (21). Other cells such as tumor-associated macrophages (TAMs), neutrophils and regulatory T-cells (Tregs) are recruited to these pre-metastatic sites and lead to impaired T-cell activation and further dampening of immune surveillance (22). In addition to immune suppression, neutrophils infiltrate the PMN and adopt protumor phenotypes, releasing neutrophil extracellular traps (NETs) and proteases that remodel the stroma and further support tumor cell adhesion and survival (23). Stromal fibroblasts and resident mesenchymal cells are activated by exosomal signals to express pro-tumorigenic mediators and drive ECM reorganization, including fibronectin deposition and matrix metalloproteinase (MMP) production, to form a scaffold that enhances recruitment and retention of supportive cells and facilitates circulating tumor cell extravasation and engraftment (16).

Tumor-derived exosomal microRNAs can also directly affect endothelial integrity in distant organs. Exosomal miR-25-3p targets the transcription factors KLF2 and KLF4 in endothelial cells, leading to reduced expression of tight junction proteins including ZO-1, occludin, and claudin-5. This disruption of endothelial barriers increases vascular permeability and promotes angiogenesis, facilitating the extravasation of circulating tumor cells and supports the formation of PMNs in both lung and liver (12).

Although many of these mechanisms are broadly shared across metastatic sites, the immune composition and stromal architecture of the PMN vary substantially between organs. In CRC, the liver and lung represent the most common metastatic sites, yet they develop distinct niche environments shaped by their unique immune landscapes and resident stromal populations.

## 3 Organ-specific metastatic niche formation and immune landscapes

**Figure 1:**
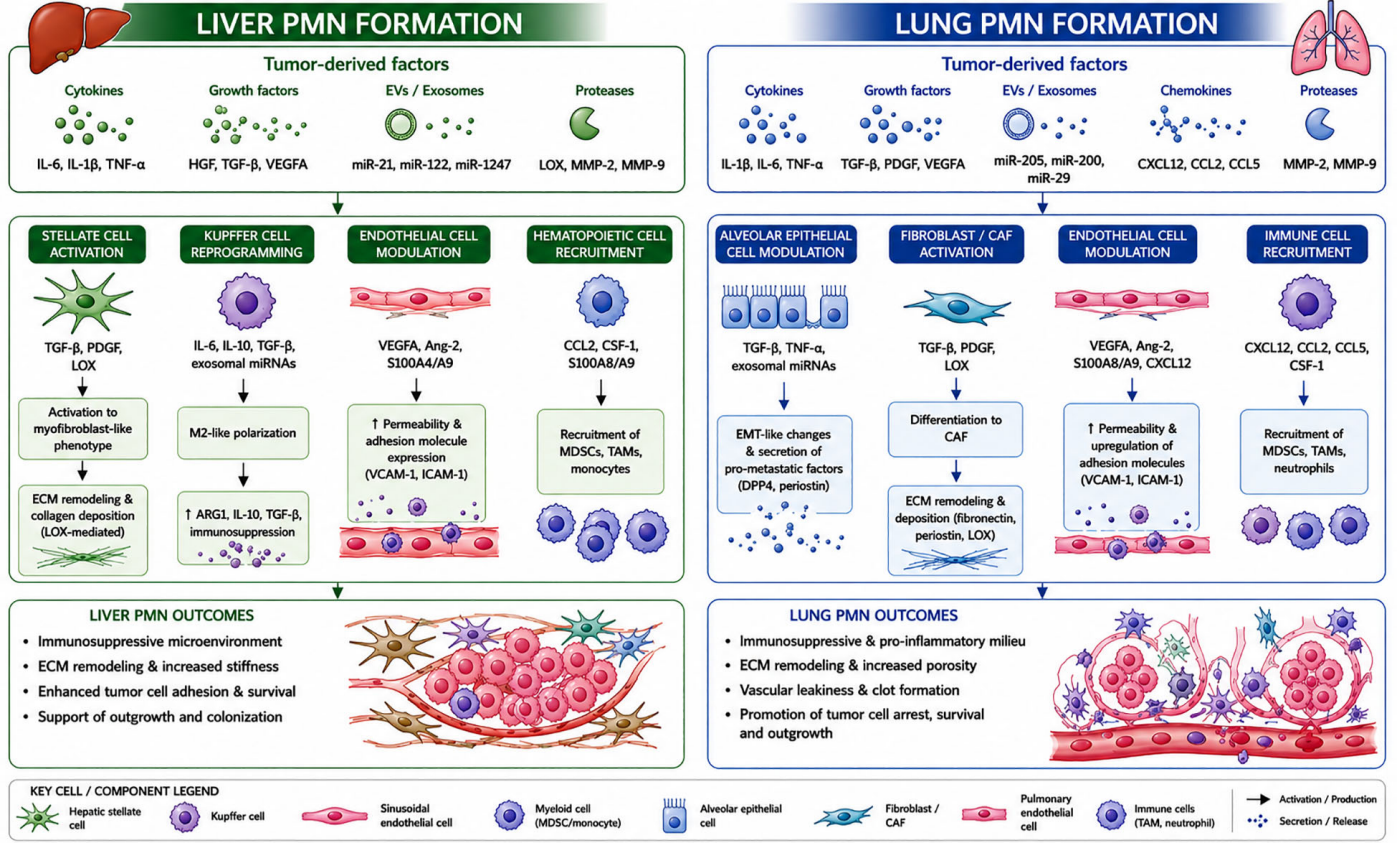
Premetastatic-niche formation in the liver vs lung. Ang-2, angiopoietin-2; ARG1, arginase 1; CAF, cancer-associated fibroblast; CCL2, C-C motif chemokine ligand 2; CCL5, C-C motif chemokine ligand 5; CSF-1, colony-stimulating factor 1; CXCL12, C-X-C motif chemokine ligand 12; ECM, extracellular matrix; EMT, epithelial–mesenchymal transition; EVs, extracellular vesicles; HGF, hepatocyte growth factor; ICAM-1, intercellular adhesion molecule 1; IL-1β, interleukin 1 beta; IL-6, interleukin 6; IL-10, interleukin 10; IL-11, interleukin 11; LOX, lysyl oxidase; MDSCs, myeloid-derived suppressor cells; miR, microRNA; MMP-2, matrix metalloproteinase 2; MMP-9, matrix metalloproteinase 9; PDGF, platelet-derived growth factor; PMN, pre-metastatic niche; SPP1, secreted phosphoprotein 1; TAMs, tumor-associated macrophages; TANs, tumor-associated neutrophils; TGF-β, transforming growth factor beta; TNF-α, tumor necrosis factor alpha; VCAM-1, vascular cell adhesion molecule 1; VEGFA, vascular endothelial growth factor A.

CRC pre-conditions the microenvironment of the liver and lung through distinct but overlapping mechanisms. **Figure 1** summarizes the PMN formation in the liver and lung.

### 3.1 Liver

In CRC, the liver represents the most common site of metastasis. Beyond its anatomical exposure to tumor cells through the portal circulation, the liver provides a permissive microenvironment that facilitates early PMN formation. The liver is intrinsically a tolerogenic organ due to its continuous exposure to antigens, microbial products, and metabolites derived from the gut. To prevent excessive inflammation against these largely harmless stimuli, the hepatic immune system is biased toward immune restraint rather than full effector activation (24, 25). This tolerogenic state is maintained by multiple resident hepatic cell populations, including Kupffer cells, liver sinusoidal endothelial cells, stellate cells, and other antigen-presenting cells, which favor deletion or dysfunction of activated T-cells, induction of regulatory pathways, and local production of suppressive mediators (25). Tumor-derived EVs and soluble factors released by the primary tumor further remodel the already immune-tolerant hepatic immune and stromal landscape to support metastatic seeding (26).

Exosomal microRNA−934 is an important mediator of liver metastasis in CRC. Higher levels are associated with higher T and M stages and in the presence of liver metastasis. Increased levels are also associated with poor survival outcomes (27). Exosomal microRNA−934 induces macrophage polarization toward the immunosuppressive M2 phenotype via PTEN/PI3K–AKT signaling (27). Similarly, EVs enriched in heat shock protein 90B1 (HSP90B1) promote polarization of hepatic macrophages from a pro-inflammatory M1 phenotype toward an M2 phenotype while reducing CD8^+^ T-cell viability, thereby contributing to the already tolerogenic hepatic niche (28). In turn, M2 TAMs promote further immunosuppression and TGF-β production (29). Other EV cargos further enhance metastatic potential: the metalloproteinase ADAM17, which is enriched in CRC exosomes, promotes tumor cell migration through cleavage of E-cadherin and facilitates liver metastasis (30), and EV-associated microRNA-21 activates Toll-like receptor-7 (TLR7) signaling in hepatic macrophages, triggering interleukin-6 production and driving expansion of myeloid-derived suppressor cells (MDSCs), which suppress T-cell responses (26, 31). Natural killer (NK) cell cytotoxicity can also be impaired through TGF-β1 delivered within tumor-derived EVs (32).

In parallel with EV signaling, CRC tumors secrete soluble factors that recruit immunosuppressive myeloid populations to the liver. Tumor-derived vascular endothelial growth factor-A (VEGF-A) stimulates tumor-associated macrophages to produce CXCL1, which accumulates in pre-metastatic hepatic tissue and recruits CXCR2^+^ MDSCs to the developing niche (33). Similarly, CRC-associated tissue inhibitor of metalloproteinases-1 (TIMP-1) increases hepatic stromal-derived factor-1 (SDF-1) levels to promote neutrophil recruitment to the liver (34). These infiltrating myeloid populations represent key cellular components of the hepatic PMN.

Stromal remodeling is another critical step in hepatic niche formation. Hepatic stellate cells (HSCs), the major fibroblastic cells of the liver, can be activated by CRC-derived exosomes enriched in TGF-β1 and miR-188-3p. This activation drives their transition toward a cancer-associated fibroblast (CAF) phenotype (32, 35). Activated HSC-derived CAFs secrete ECM proteins such as fibronectin, leading to remodeling of the hepatic stroma and the generation of an adhesive scaffold that facilitates tumor cell retention and colonization. These CAFs also produce chemokines, including CXCL12, which further recruit immunosuppressive myeloid cells and reinforce niche development (35).

The resulting hepatic PMN is characterized by the accumulation of immunosuppressive myeloid cells and depletion of CD8^+^ T-cells (28, 36). This reflects the intrinsically tolerogenic immune environment of the liver. Tumor-derived signals amplify macrophage-driven immune suppression, stellate cell activation, and extracellular matrix remodeling, ultimately generating a microenvironment that supports metastatic colonization.

### 3.2 Lung

In contrast to the liver, the lung PMN in CRC is generally characterized by stronger inflammatory signaling and pronounced recruitment of neutrophils and other myeloid populations. Tumor-derived factors and organ-tropic exosomal molecules prime pulmonary tissues, creating an inflammatory microenvironment that facilitates tumor cell extravasation and colonization (37). Tumor-secreted factors such as CRC-derived VEGF-A can stimulate tumor-associated macrophages to release CXCL1, which mobilizes CXCR2^+^ MDSCs to the lung (22). Once recruited, these MDSCs suppress antitumor immunity through the production of TGF-β, reactive oxygen species, and interleukin-10, while also promoting regulatory T-cell differentiation and inhibiting natural killer and CD8^+^ T-cell function (38). These processes contribute to a microenvironment that contains suppressed T-cells.

Tumor-derived molecules also contribute to pulmonary niche formation by activating resident lung cells. Exosomal RNAs can stimulate Toll-like receptor-3 (TLR3) signaling in alveolar epithelial cells, which induces the secretion of chemokines such as CXCL5 and CXCL12 that recruit neutrophils to the lung (22, 39). In parallel, CRC-derived EVs containing integrin β-like 1 (ITGBL1) or microRNAs such as miR-146 and miR-155 are taken up by lung fibroblasts, activating NF-κB and STAT3 signaling pathways. This signaling converts fibroblasts into CAF-like cells that secrete inflammatory cytokines including IL-6, TNF-α, TGF-β, and CXCL12, thereby amplifying inflammatory signaling within the pulmonary stroma (38, 40). Tumor-derived factors also disrupt pulmonary vascular integrity, facilitating tumor cell extravasation. VEGF-A–driven inflammatory signaling can stimulate prostaglandin E_2_ (PGE_2_) production to promote endothelial activation and immune cell infiltration (22).

Recruited neutrophils play a particularly important role in lung niche development. Tumor exosomal RNAs activate alveolar epithelial TLR3, inducing chemokine production and recruiting neutrophils (39). Under the influence of cytokines such as IL-6, IL-10, and granulocyte colony-stimulating factor (G-CSF), neutrophils can acquire a pro-tumorigenic “N2” phenotype. These N2 neutrophils release MMPs and damage-associated proteins such as S100A8/A9, which remodel the extracellular matrix and recruit additional immune cells to the niche (38, 41). Furthermore, neutrophils contribute to vascular destabilization through the release of proteases such as neutrophil elastase, which can damage endothelial barriers and promote PMN formation (42). Pre-clinical studies have demonstrated the critical role of neutrophils in driving lung metastasis in CRC specifically. One pre-clinical study showed that the loss of SMAD4 (which occurs in 20-40% of cases), promotes lung metastasis by accumulation of CCR1^+^ tumor-associated neutrophils (TANs) through the CCL15-CCR1 axis, and that CCL15 expression by metastatic cancer cells confers worse prognosis (43). Another pre-clinical study showed that after insufficient radiofrequency ablation of liver metastasis from CRC, residual tumor cells recruit CD177^hi^ PAD4^hi^ neutrophils and generate NETs that accelerate lung metastasis through MEK/ERK signaling (44).

Finally, activated lung CAFs remodel the pulmonary stroma by depositing extracellular matrix components, including fibronectin, collagen, and periostin. These structural changes generate a supportive matrix that facilitates tumor cell adhesion and survival within the lung microenvironment (38).

In contrast to the tolerogenic liver niche, lung PMN formation is largely driven by inflammatory signaling and neutrophil-dominated immune recruitment. These processes promote endothelial activation, vascular permeability, and inflammatory stromal remodeling, collectively generating a pulmonary environment permissive for metastatic colonization.

## 4 Mechanisms of response to immune checkpoint inhibition within the TME

After the formation of the pre-metastatic niche, circulating tumor cells attempt to establish secondary tumors in distant organs. For the tumor cells, the process is highly inefficient, and only about 0.01% of circulating tumor cells are successful (45). Once metastasis is established, immune escape and resistance to ICIs are driven by different processes within the lung and the liver.

### 4.1 Isolated liver metastasis

Liver metastasis is a major negative modifier of ICI outcomes in CRC, even in tumors that are otherwise genomically favorable (MSI-H/dMMR) (46). This reflects the unique immunosuppressive landscape of the liver, which can limit PD-1/PD-L1 efficacy through both local and systemic mechanisms (46).

Within the liver, stromal and myeloid circuits reinforce immune exclusion. SPP1^high^ TAMs in close proximity to FAP^+^ CAFs at the invasive margin form stromal–myeloid niches that exclude T-cells, reduce antigen presentation, and upregulate checkpoint ligands, thus establishing an immune-excluded architecture that correlates with poor clinical outcomes (47). Additionally, tumor-derived EVs containing miR-21-5p and miR-181a-5p activate macrophage TLR7 signaling, triggering IL-6 production and STAT3-mediated myeloid shift, leading to an environment that exhibits reduced antigen presentation and upregulation of PD-L1, ARG1, and IL-10, further antagonizing T-cell reactivation (26, 48).

Stromal and vascular remodeling add additional layers of resistance. CRC EVs containing TGF-β1 and miR-188-3p activate HSCs to become CAFs, which in turn promote a collagen-rich ECM that physically blocks CD8^+^ T-cell infiltration via TGF-β–dependent mechanisms (49). Tumor EVs and soluble factors also disrupt vascular integrity: miR-25-3p targets endothelial KLF2/KLF4, and ADAM17 disrupts VE-cadherin, increasing permeability but mostly favoring suppressive myeloid trafficking and dysfunctional immune infiltration rather than anti-tumor cells (12, 50, 51). Platelet-derived PD-L1 further reinforces T-cell exhaustion, particularly in liver microvasculature (52).

However, in addition to local resistance of the tumor to T-cells, systemic CD8^+^ T-cell loss is a central feature in patients with liver metastasis. Hepatic macrophages (FasL^+^ CD11b^+^ F4/80^+^) induce apoptosis of Fas^+^ CD8^+^ T-cells via Fas–FasL interactions, which siphon and deplete the T-cell populations that are essential for ICI efficacy (53). Furthermore, scarcity of dendritic cells is another feature of liver metastasis which contributes to the resistance to immune checkpoint blockade in mouse models, while such resistance was not seen with subcutaneous tumor implants (54). Therefore, the depletion of CD8^+^ T-cells leads to a diminished response to ICIs not only in the liver metastasis, but also systemically, impairing antitumor immunity across the entire body. Notably, in pre-clinical studies, liver-directed radiotherapy has been shown to deplete immunosuppressive hepatic macrophages, thereby enhancing the survival of hepatic T-cells, and decreases the sequestration of T-cells within the liver (53). Consequently, combining liver-directed radiotherapy with immunotherapy may help restore systemic antitumor immune responses (53).

In addition to systemic depletion of lymphocytes, liver metastasis affects ICI efficacy at other metastatic sites by other mechanisms. Liver metastasis induces a CTLA-4^Hi^ Treg–dependent immunosuppression, which can render anti-PD-1 monotherapy ineffective unless Tregs are destabilized or depleted with CTLA-4 blockade (55). Also, liver metastases can amplify systemic immunosuppression by licensing neutrophils through IL-1–dependent signaling, promoting neutrophil recruitment and activation (56), and by promoting a phenotype of MDSCs (distinct from that seen in lung metastasis) that diminishes immune responses (57).

Intriguingly, response to ICIs can be time-dependent. In mouse models, T-cell accumulation was detected early in the liver and tumor-draining lymph nodes, however, at later time points, mice bearing liver metastasis exhibited a systemic reduction of T-cells across all examined sites (53). This indicates that the “best window” for PD-1 blockade is prior to the development of liver metastasis, which leads to exhaustion and depletion of T-cells in the TME (53, 58).

### 4.2 Isolated lung metastasis

Patients with isolated lung metastasis do not exhibit the same immunotherapy resistance (especially systemic resistance) that is seen in patients with liver metastasis (55). Lung metastatic niche is enriched with T-cells that express higher levels of activation markers (such as CD27, CD44, ICOS, 4-1BB, and CD45RO), a greater density of antigen-presenting cells and more extensive lymphoid aggregates (59). The lung TME also exhibits higher activation of the cGAS–STING pathway, which promotes dendritic cell activation, type I interferon production, tertiary lymphoid structure formation, and enhanced cytotoxic responses from T-cells and NK cells (59). The presence of tertiary lymphoid structures is associated with a more inflamed tumor microenvironment and correlates with increased infiltration of cytotoxic T-cells and B cells (60). However, despite this immune activation, the lung TME also exhibits immunosuppressive features, largely driven by neutrophils. Within the TME, the pro-metastatic N2 neutrophils inhibit T-cell responses through CD80/CD86–CTLA-4 signaling, in addition to upregulation of PD-L1 (41). Therefore, in isolated lung metastasis, the TME contains all the components for a functional immune response: antigen-presenting cells, interferon signaling, and most importantly functional (but restrained) T-cells, which may help explain the relatively greater sensitivity of lung-only disease to ICI-based combinations (41, 59, 61).

### 4.3 Concurrent liver and lung metastasis

The coexistence of liver and lung metastases in CRC deserves specific attention because these are the 2 most common metastatic sites, yet they appear to influence prognosis and immunotherapy response differently. Population-based studies suggest that simultaneous synchronous liver and lung metastases are present in about 3.2% of all CRC cases at diagnosis, indicating that this pattern is uncommon but clinically meaningful (62). Data suggest that when both sites are present, the liver is likely the more important determinant of outcome with ICI-based therapy, because hepatic involvement is repeatedly associated with lower response rates and shorter progression-free survival in both MSS/pMMR and dMMR/MSI-H disease, whereas lung-only disease is associated with more favorable responses (46, 63, 64). Therefore, in the presence of liver metastasis, the systemic T-cell depletion and the Treg–dependent immunosuppression reduced the efficacy of ICIs (especially anti-PD-1/PD-L1 monotherapy) even in extrahepatic sites of metastasis (55). Co-occurrence of lung and liver metastases argues for a multidisciplinary strategy that does not treat all metastatic sites as biologically equivalent; instead, treatment planning may need to account for the liver as a potential driver of systemic immune resistance while still considering local therapy in carefully selected patients with limited disease burden.

## 5 TME targeting to overcome resistance to immunotherapy: clinical evidence

Across multiple MSS/pMMR CRC cohorts treated with PD-1/PD-L1–based combinations, objective responses and prolonged disease control are disproportionately observed in patients with lung-only (or predominantly lung) metastatic disease, suggesting that the lung metastatic microenvironment is, on average, less systemically tolerizing than the liver (58, 65). A systematic review evaluating the combination of ICIs with chemotherapy or targeted therapy demonstrated that in different trials, the response in lung lesions is generally higher than that in liver lesions (8). **Table 1** summarizes the studies evaluating immunotherapy-based strategies in metastatic CRC.

**Table 1:** Studies evaluating immunotherapy-based strategies in metastatic colorectal cancer.

Trial/study	Regimen	Population	Key result/site-specific finding
KEYNOTE-177 (68) (Phase III)	Single-agent ICI: Pembrolizumab vs chemotherapy	First-line MSI-H/dMMR mCRC	Metastatic-site outcomes not reported
Chen et al. (66) (Phase II)	Dual ICI: Durvalumab + tremelimumab vs BSC	Refractory MSS/pMMR mCRC	DCR 49% without liver metastasis vs 14% with liver metastasis
Bullock et al. (67) (Phase I)	Dual ICI: botensilimab + balstilimab	Heavily pretreated MSS/pMMR mCRC	ORR 23%, DCR 80%, median OS 20.9 months in patients without liver metastasis
CheckMate 8HW (69, 70) (Phase III)	Dual ICI: Nivolumab + ipilimumab vs nivolumab or chemotherapy	MSI-H/dMMR mCRC	Dual ICI improved outcomes overall; PFS benefit vs nivolumab was stronger without liver metastasis
KEYFORM-007 (71) (Phase III)	Dual ICI: Favezelimab + pembrolizumab vs SOC	Previously treated MSS/pMMR, PD-L1–positive mCRC	Failed OS endpoint; liver-metastasis subgroup not reported
RELATIVITY-123 (72, 73) (Phase III)	Dual ICI: Nivolumab + relatlimab vs SOC	Later-line mCRC	Terminated early; did not meet OS endpoint
Checkmate 142 (74) (Phase II)	Dual ICI: Nivolumab+relatlimab	Previously treated MSI-H/dMMR mCRC	ORR 39% with liver metastasis vs 59% without liver metastasis
AtezoTRIBE (75) (Phase II)	ICI + anti-VEGF+ chemotherapy: FOLFOXIRI + bevacizumab ± atezolizumab	First-line mCRC; mostly MSS/pMMR	No clear PFS benefit by liver-only status
Wang et al. (76) (Phase II/III)	ICI + anti-VEGF+ chemotherapy: Serplulimab + HLX04 + XELOX vs placebo + HLX04 + XELOX	First-line mCRC	Liver metastasis did not significantly affect OS
CAPABILITY-01 (77) (Phase II)	ICI + anti-VEGF+epigenetic therapy: Sintilimab + chidamide ± bevacizumab	Later-line MSS/pMMR mCRC	Triplet improved outcomes in patients with liver metastasis
Johnson et al. (78) (Phase II)	ICI + MEK inhibitor: Durvalumab + trametinib	MSS/pMMR mCRC	ORR 3.5%; lung lesions showed better control than liver lesions in mixed-site patients
Lentz et al. (79) (Phase II)	ICI + MEK inhibitor + anti-VEGF: Pembrolizumab + binimetinib + bevacizumab	Refractory MSS/pMMR mCRC	ORR 25% without liver metastasis vs 7.9% with liver metastasis
Fakih et al. (80) (Phase I)	ICI + TKI: Regorafenib + ipilimumab + nivolumab	Later-line MSS/pMMR mCRC	ORR 36.4% without liver metastasis; 0% with liver metastasis
REGONIVO (81) (Phase Ib)	ICI + TKI: Regorafenib + nivolumab	Advanced gastric cancer and MSS/pMMR mCRC	CRC ORR 36%; ORR 50% in lung metastasis vs 15.4% in liver metastasis
Fakih et al. pooled analysis (65)	ICI + TKI: Regorafenib + nivolumab ± ipilimumab	MSS/pMMR mCRC	ORR 56.3% in lung-only metastasis vs 0% with liver/peritoneal metastasis
CAMILLA (82) (Phase II)	ICI + TKI: Durvalumab + cabozantinib	Refractory GI cancers; MSS/pMMR CRC	MSS CRC ORR 27.6%; responses occurred in some patients with liver metastasis
LEAP-017 (83) (Phase III)	ICI + TKI Lenvatinib + pembrolizumab vs SOC	Previously treated MSS/pMMR mCRC	OS benefit favored patients without liver metastasis
STELLAR-303 (84) (Phase III)	ICI + TKI: Zanzalintinib + atezolizumab vs regorafenib	Refractory MSS/pMMR mCRC	OS benefit seen with and without liver metastasis
EORTC-1560-GITCG/ILOC (85) (Phase II)	ICI + local liver therapy: Liver RFA/SBRT + durvalumab + tremelimumab	Unresectable liver-predominant mCRC	No objective responses in untreated lesions; median PFS 2.2 months
Ge et al. (86) (Translational)	ICI + local therapy: RT + ICI	MSS/pMMR mCRC	Liver metastases promoted systemic myeloid suppression despite local immune activation
MEDIPLEX (87) (Phase I)	Macrophage/CSF1R targeting + ICI: Pexidartinib + durvalumab	Advanced pancreatic cancer and CRC	Clinical benefit at 2 months was 21%, all stable disease
Mahalingam et al. (88) (Phase I)	Macrophage/TME targeting: Imalumab	Advanced solid tumors including mCRC	Stable disease in 13/39 evaluable patients; no objective responses
Le et al. (89) (Phase Ib/II)	CCR2/CCR5 targeting ± ICI: BMS-813160 + chemotherapy ± nivolumab	Advanced pancreatic cancer and MSS/pMMR mCRC	In mCRC, ORR was not improved over FOLFIRI alone
Halama et al. (90) (Phase I/II)	CXCL12 targeting ± ICI: NOX-A12 ± pembrolizumab	Heavily pretreated MSS/pMMR mCRC and pancreatic cancer	No objective responses; 25% stable disease in combination phase
Bendell et al. (91) (Phase I)	CD73/adenosine targeting ± ICI: Oleclumab ± durvalumab	Advanced solid tumors including CRC	Low activity; CRC ORR 2.4% in expansion cohort
Yamazaki et al. (92) (Phase II)	TGF-β targeting + chemoradiotherapy: Galunisertib + neoadjuvant chemoradiotherapy	Locally advanced rectal adenocarcinoma	Complete response in 32%; not metastatic CRC-specific
REGAL-1 (93) (Phase Ib)	Angiogenesis/ALK1 targeting + TKI: PF-03446962 + regorafenib	Refractory mCRC	Minimal activity; one patient achieved stable disease
Tolcher et al. (94) (Phase I)	TGF-β receptor targeting: LY3022859	Advanced solid tumors	No responses observed

ALK1, activin receptor-like kinase 1; BSC, best supportive care; CRC, colorectal cancer; DCR, disease control rate; ICI, immune checkpoint inhibitor; mCRC, metastatic colorectal cancer; MEK, mitogen-activated protein kinase kinase; MSS, microsatellite stable; ORR, objective response rate; OS, overall survival; PFS, progression-free survival; pMMR, proficient mismatch repair; RFA, radiofrequency ablation; RT, radiotherapy; SBRT, stereotactic body radiotherapy; SOC, standard of care; TKI, tyrosine kinase inhibitor; TME, tumor microenvironment.

### 5.1 ICI combinations

A phase 2 study randomized 180 patients with metastatic MSS CRC to receive either dual ICIs with durvalumab (anti-PD-L1) and tremelimumab (anti-CTLA4) or best supportive care. One hundred and nineteen patients received dual ICI, of which 80 (67.2%) had liver metastasis. In the dual ICI arm, the disease control rate (DCR) was 49% in patients without liver metastasis compared to 14% in patients with liver metastasis (p = 0.03) (66). A phase 1 trial evaluated botensilimab (anti-CTLA-4) plus balstilimab (anti-PD-1) in 87 heavily pretreated patients with MSS metastatic CRC. Among the 69 patients without liver metastasis, the ORR was 23%, the DCR was 80%, and the median OS was 20.9 months (67). Regarding MSI-H/dMMR metastatic CRC, while the KEYNOTE-177 that evaluated single-agent pembrolizumab did not report outcomes by metastatic site, a retrospective review of patients who received single agent pembrolizumab revealed that patients with liver metastasis had significantly worse PFS compared to those without liver metastasis (HR 2.6, P = 0.003) (64, 68). In the phase III CheckMate 8HW trial, which included three arms (dual immune checkpoint inhibition with ipilimumab plus nivolumab, nivolumab monotherapy, and chemotherapy), the ICI combination demonstrated statistically significant improvement regardless of metastatic site (liver, lung, and peritoneum) compared to chemotherapy. However, subgroup analysis showed that compared to nivolumab monotherapy, the use of dual ICI has led to a significant improvement in PFS only in the absence of liver metastasis (69, 70).

The KEYFORM-007 is a phase 3 trial that evaluated the efficacy of favezelimab (anti–LAG-3 antibody) in combination with pembrolizumab (anti-PD-1) compared to standard of care (regorafenib or TAS-102) in patients with MSS/pMMR CRC that progressed after treatment with chemotherapy. Patients were stratified by the presence of liver metastasis, however, the trial failed to meet its primary endpoint of improvement of OS. Results by the presence of liver metastasis are not yet reported (71). Similarly, the RELATIVITY-123, a phase 3 trial investigating nivolumab (anti PD-1) + relatlimab (anti–LAG-3 antibody) compared to standard of care (regorafenib or TAS-102) in later-line metastatic mCRC (mCRC) was terminated early due to inability to meet its primary endpoint of OS (72, 73). The CheckMate 142 is another trial (phase II) evaluating the combination of nivolumab (anti-PD-1) and relatlimab (anti-lymphocyte-activation gene 3 (LAG-3)) in patients with MSI-H/dMMR metastatic CRC previously treated with chemotherapy. In 50 enrolled patients, the ORR was 50% and DCR was 70%. In 18 patients with liver metastasis and 32 patients without liver metastasis, the ORR was 39% and 59% respectively (74).

### 5.2 ICI and anti-VEGF combinations

The ATEZOTRIBE is a randomized phase 2 trial in which patients with metastatic CRC received FOLFOXIRI plus the anti-VEGF bevacizumab, with or without atezolizumab (anti-PD-L1). The majority of the patients (91-92%) had MSS/pMMR CRC. In the subgroup analysis, the addition of atezolizumab did not significantly affect progression-free survival (PFS) regardless of the presence or absence of liver-only metastasis. This indicates that anti-VEGF therapy may improve outcomes in patients with liver metastasis, potentially mitigating the adverse prognostic impact of hepatic involvement. This is likely due to the normalization of tumor vasculature, leading to enhanced CD8^+^ T-cell infiltration (75). Likewise, another randomized phase 2 trial that evaluated the combination of HLX04 (an approved bevacizumab biosimilar) and XELOX, with or without serplulimab (anti-PD-1). The presence of liver metastasis did not have any significant effect on OS (76).

The CAPABILITY-01 trial is a randomized phase 2 trial that evaluated the efficacy of sintilimab (anti-PD-1) with chidamide (histone deacetylase inhibitor) with or without bevacizumab (anti-VEGF) in patients with MSS/pMMR metastatic CRC in later-line of treatment. 48 patients were enrolled; 26 patients had liver metastasis and 33 patients had lung metastasis. 23 patients were randomized to the doublet arm and 25 patients to the triplet arm. Among the 26 patients with liver metastasis, the 18-week PFS was higher in the triplet arm compared to the doublet arm (64.3% versus 8.3% respectively, P = 0.005) and a longer median PFS of 7.3 months versus 1.4 months respectively, (P = 0.001), in addition to better ORR of 50.0% versus 8.3% respectively (P = 0.036) and better DCR of 71.4% versus 8.3% respectively, (P = 0.002). In the triplet arm, the presence and absence of liver metastasis demonstrated numerically similar outcomes regarding PFS, ORR and DCR, while in the doublet arm, patients with liver metastasis had displayed numerically lower outcomes compared to patients without liver metastasis (18-week PFS 8.3% versus 36.4%, median PFS 1.4 months versus 3.7 months, ORR 8.3% versus 18.2%, DCR 8.3% versus 72.7%, respectively) (77).

### 5.3 ICI and tyrosine kinase inhibitor (TKI) combinations

A phase II trial by Johnson et al. evaluated durvalumab (anti-PD-L1) in combination with trametinib (MEK inhibitor) in MSS metastatic CRC. In this cohort, there was one patient with liver-only metastasis, 4 patients with lung-only metastasis, and 6 patients had metastasis to both sites. The ORR was 3.5% (1/29 patients had partial response). Regarding response by disease site, in six patients with both liver and lung metastasis, the lung lesions showed good response in one patient and stable disease in the other 2 patients, while the liver lesions showed stable disease in one patient and progression in the two other patients. The responses in the remaining patients were not mentioned (78). Another phase II trial of the combination of pembrolizumab with binimetinib and bevacizumab including 50 patients with MSS metastatic CRC demonstrated an ORR of 12% and a DCR of 70%, with a trend toward higher ORR in 3/12 patients without liver metastasis (25%) compared to 3/38 patients with liver metastasis (7.9%) (79).

The combination of regorafenib, ipilimumab (anti-CTLA4), and nivolumab (anti-PD-1) was investigated in a phase 1 trial 3 + 3 dose de-escalation study in patients with MSS/pMMR metastatic CRC in later line of treatment. Of the 39 enrolled patients, the ORR was 27.6% with a median PFS of 4 months, and a median OS of 20 months. In 22 patients without liver metastasis, the ORR was 36.4%. All patients with liver metastasis did not respond to treatment (80). In the REGONIVO trial that investigated regorafenib combined with nivolumab (anti-PD-1) (without chemotherapy) in later line in patients with MSS metastatic colorectal and gastric cancers, response occurred in 36% (9/25 patients) in CRC. The ORR in patients with lung metastasis was 50% (8/16 patients) while in liver metastasis it was 15.4% (2/13 patients) (81). A pooled analysis of patients in these 2 trials who were treated with regorafenib+nivolumab (± ipilimumab) showed an ORR of 0% in patients with liver or peritoneal metastasis vs 56.3% in lung-only metastasis (65).

In contrast, in the phase 2 basket trial CAMILLA investigating durvalumab+cabozantinib in refractory GI cancers, the MSS CRC cohort (29 patients) showed a 27.6% ORR (8/29 patients) including 4/23 patients with liver metastasis (82).

The LEAP-017 trial is a phase 3 trial in which patients with MSS/pMMR metastatic CRC who progressed after treatment with chemotherapy, were randomized to receive either lenvatinib plus pembrolizumab (anti-PD-1) or standard of care (regorafenib or trifluridine/tipiracil). Patients were stratified by the presence of liver metastasis: in the 241 patients in the experimental arm, 168 patients (70%) had liver metastasis. OS was significantly higher in patients without liver metastasis with a hazard ratio (HR) of 0.65 [95% CI, 0.42 to 0.99] (83).

The STELLAR-303 is a phase 3 trial that investigated the efficacy of the multitargeted TKI zanzalintinib plus atezolizumab versus regorafenib in patients with MSS/pMMR CRC who progressed after prior lines of chemotherapy. In this trial, patients were also stratified by the presence of liver metastasis: among 451 patients in the zanzalintinib plus atezolizumab arm, 264 patients (59%) had liver metastasis. The use of zanzalintinib plus atezolizumab resulted in significantly better OS in patients with and without liver metastasis compared to regorafenib (84).

### 5.4 Anti-PD-1/PD-L1 and local treatment combinations

The EORTC-1560-GITCG single-arm phase II study evaluated the combination of partial local treatment of the liver (2 liver lesions or one liver lesion and one extrahepatic lesion should remain untreated) using radiofrequency ablation or stereotactic body radiotherapy with dual immunotherapy (tremelimumab plus durvalumab) in patients with unresectable, liver-predominant metastatic CRC who had at least stable disease after prior chemotherapy. Of the 20 patients who received treatment, no objective responses were observed in the untreated metastasis, with the best response being stable disease in 42.9% of patients, and a median PFS of 2.2 months (85). Additionally, a study by Ge et al. demonstrated that in MSS/pMMR metastatic CRC, the efficacy of ICIs is profoundly limited by the metastatic microenvironment, especially in the liver. Despite evidence of local immune activation following treatment, liver metastasis promotes a systemic immunosuppressive state characterized by expansion of suppressive myeloid populations, ultimately blunting effective T-cell–mediated anti-tumor responses and contributing to resistance to ICIs (86).

### 5.5 Targeting other components of the TME

MEDIPLEX was a phase I dose-escalation and expansion study evaluating pexidartinib (CSF1R inhibitor targeting TAMs) in combination with durvalumab (anti–PD-L1) in patients with advanced/metastatic pancreatic cancer and CRC. A total of 19 patients were enrolled in the dose-escalation phase, followed by expansion cohorts including 14 CRC patients. Clinical benefit at 2 months was 21% (all stable disease), with more durable benefit observed only in MSI-H CRC patients, indicating limited activity of this TME-targeting strategy in unselected populations (87).

Another phase I dose-escalation study evaluated imalumab (which is an anti–oxidized macrophage migration inhibitory factor antibody targeting the TME) in patients with advanced solid tumors, including metastatic CRC. A total of 50 patients received treatment. Antitumor activity was limited, with stable disease observed in 13 of 39 evaluable patients and no objective responses reported (88).

Another phase Ib/II trial evaluated BMS-813160 (a dual CCR2/CCR5 antagonist targeting myeloid cell recruitment in the TME) in combination with chemotherapy ± nivolumab in patients with advanced pancreatic cancer and mCRC. Across both parts, over 140 patients were enrolled, including cohorts with second-line MSS metastatic CRC. In metastatic CRC, ORR was 19% (BMS-813160 + FOLFIRI), 13% (lower dose + FOLFIRI), compared to 27% with FOLFIRI alone (89).

A phase 1/2 open-label study evaluated NOX-A12 (CXCL12 inhibitor) monotherapy and in combination with pembrolizumab in heavily pretreated patients with MSS/pMMR metastatic CRC and pancreatic cancer. NOX-A12 monotherapy induced Th1 cytokines (IFNγ, IL-2, IL-16) in approximately half of patients, though no objective responses were observed. In the combination phase, 25% of patients achieved stable disease, and 35% showed prolonged time on treatment compared to prior therapy. Median PFS was 1.87 months, with OS rates of 42% at 6 months and 19% at 12 months (90).

A phase I dose-escalation and expansion study evaluated oleclumab (an anti-CD73 monoclonal antibody that blocks CD73-mediated adenosine production to reduce local tumor immunosuppression) alone or in combination with durvalumab in patients with solid tumors including advanced CRC. Objective responses were limited: 0% in escalation, and 2.4% (CRC), 4.8% (PDAC), and 9.5% (NSCLC) in expansion cohorts, with 6-month PFS rates of 5.4%, 13.2%, and 16.0%, respectively (91).

A phase 2 single-arm study evaluated the addition of galunisertib (a TGF-β type I receptor kinase inhibitor that blocks immunosuppressive TGF-β signaling) to standard neoadjuvant chemoradiotherapy in patients with previously untreated, locally advanced rectal adenocarcinoma (stage IIA–IIIC/IV). Among 38 enrolled patients, 12 (32%) achieved a complete response (including pathological complete responses in 7 patients). Additionally, clinical complete responses treated with watch and wait were maintained at 1 year in 5 patients (92).

A Phase I, dose-escalation study evaluated PF-03446962 (a monoclonal antibody targeting activin receptor-like kinase 1 to inhibit tumor angiogenesis) in combination with regorafenib, mCRC. Seventeen patients were enrolled (11 evaluable). Only one patient achieved stable disease for 5.5 months but discontinued due to toxicity (93).

A phase I study evaluated LY3022859 (an anti-TGFβRII IgG1 monoclonal antibody that inhibits TGF-β receptor–mediated signaling) in patients with advanced solid tumors. Fourteen patients were enrolled across three cohorts and no responses were seen (94).

## 6 Strategies to prevent metastasis formation in high-risk localized CRC

### 6.1 Exosome–integrin blockade

As mentioned previously, tumor-derived exosomes carry specific integrins that direct organotropism and prime distant microenvironments. For example, CRC exosomes enriched in αvβ5 preferentially home to liver Kupffer cells, while α6β4/α6β1 patterns favor lung niche formation (19). These integrin–extracellular matrix interactions trigger Src signaling and pro-metastatic inflammation, creating a permissive PMN. Blocking integrin binding should therefore prevent exosome uptake and niche formation (19). Cilengitide, a cyclic RGD peptide that antagonizes αvβ3/αvβ5, has been studied as a prototype integrin inhibitor. *In vitro*, cilengitide reduces integrin gene expression and tumor cell proliferation (95). In CRC mouse models, integrin blockade strongly suppressed metastasis. Overexpression of TIMP1 in CRC cells drove liver metastasis via β1-integrin–Akt/mTOR signaling. Treatment with cilengitide abolished TIMP1-induced macrophage M2 polarization, reduced hepatic tumor burden, and restored overall survival to control levels. In CT26 and MC38 murine CRC models, cilengitide significantly reduced liver metastatic foci and prolonged survival (96). Currently, no CRC-specific trials of exosome/integrin inhibitors have completed.

### 6.2 NET inhibition

Due to their role in capturing tumor cells and promoting their adhesion/invasion, NETs have been studied as a potential target in the PMN (97). PAD4 is a key enzyme that citrullinates histones, enabling chromatin decondensation in NET formation. Thus, degraders of NETs (DNase I) or PAD4 inhibitors should disrupt this process in the PMN (97). Multiple pre-clinical studies implicate NETs in metastasis. In a seminal breast cancer model, inhibiting NETs (genetic or DNase I) blocked pulmonary metastasis (97). In CRC contexts, PAD4 inhibition/deficiency reduced liver metastasis in murine models (98). More recently, in CRC immunotherapy models, genetic PAD4 deletion or DNase I treatment reversed anti-PD-1 resistance through enhancement of CD8^+^ T-cell infiltration (99). To date, no clinical trials have tested NET-targeting in CRC patients.

### 6.3 Innate immune agonists (STING and TLR Ligands)

The cGAS–STING pathway enables detection of abnormal (cancerous) DNA and induces anti-tumor effects through type I interferons and pro-inflammatory cytokines, leading to recruitment of dendritic cells and T-cells (100). TLR agonists similarly stimulate myeloid cells and IFN production. In CRC models, exogenous STING or TLR ligands enhance antigen presentation and T-cell trafficking (100, 101). Intratumoral STING agonist (ADU-S100) plus a TLR9 agonist (CpG1826) had synergistic antitumor activity in a CRC model. The combination induced high levels of interferons and inflammatory cytokines, recruited CD8^+^ T-cells, and even reduced CAFs, in addition to improvement of anti-PD-1 efficacy (100, 101). There are no clinical trials evaluating this combination.

### 6.4 TGF-β and TAM inhibition

TGF-β promotes metastasis by suppressing CD8^+^ T-cell infiltration and inducing SPP1^+^ macrophages (M2 phenotype subset), thereby establishing an immunosuppressive PMN. Inhibition of TGF-β signaling has been shown to prevent metastasis formation and sensitize tumors to immune-mediated clearance, with eradication of metastatic disease in pre-clinical models when combined with checkpoint blockade (102).

CSF1R^+^ TAMs (an M2 phenotype subset) cause immunosuppression through secretion of TGF-β and other mediators and are critical for PMN formation (29). While CSF1R inhibitors (e.g., AMG 820) have entered clinical trials in CRC, these studies have largely been conducted in advanced disease rather than the adjuvant setting (87). Nevertheless, mechanistic CRC studies demonstrate that chemotherapy induces CSF1 release, which recruits CSF1R^+^ TAMs that amplify TGF-β signaling and immune evasion, thereby facilitating metastatic progression (29). Therefore, CSF1R blockade may reduce TAM infiltration, enhance CD8^+^ T-cell recruitment, and decrease metastatic burden in CRC, supporting its rationale for metastasis prevention strategies. However, the absence of dedicated adjuvant trials highlights a major translational gap.

### 6.5 Inflammation and COX-2 pathway

Chronic inflammation is a major driver of metastatic recurrence in CRC, partly through recruitment of myeloid cells and activation of pro-metastatic cytokine networks (22). Unlike most TME-targeted strategies, COX-2 inhibition has been evaluated in true adjuvant randomized trials in nonmetastatic CRC. In a meta-analysis of five randomized controlled trials (n = 7246), anti-inflammatory agents significantly improved DFS (HR = 0.85; P = .008) and prolonged time to recurrence (HR = 0.61; P = .003). However, no statistically significant benefit was observed in OS; (HR = 0.90; P = .07) or overall recurrence rate. Subgroup analysis demonstrated a more pronounced effect with aspirin, which improved DFS (HR = 0.70; P = .03), and a substantial reduction in recurrence risk among patients harboring PIK3CA mutations (HR = 0.56; P <.0001) (103).

## 7 Limitations and potential confounding factors

Although growing evidence supports the role of organ-specific immune microenvironments in shaping metastatic progression and response to immunotherapy, these clinical data should be interpreted with caution as several limitations should be considered when interpreting current findings. First, most site-specific findings are derived from exploratory subgroup analyses, retrospective cohorts, or early-phase single-arm studies rather than prospectively powered comparisons by metastatic organ. In many trials, the numbers of patients with lung-only disease, liver-only disease, or mixed metastatic patterns were small, and subgroup analyses were not adjusted for multiple comparisons. These studies may be affected by selection bias, heterogeneity in treatment regimens, and differences in prior systemic therapies, which can confound the interpretation of organ-specific treatment effects. Therefore, although the available evidence consistently suggests poorer ICI efficacy in patients with liver metastases and relatively more favorable outcomes in lung-dominant disease, future trials that prospectively stratify by metastatic site, report lesion-level responses by organ, and apply appropriate statistical correction should be conducted to confirm these findings.

Second, metastatic CRC is biologically heterogeneous. Tumor-intrinsic factors such as tumor mutational burden, neoantigen load, interferon signaling, and antigen-presentation capacity may strongly influence responsiveness to ICIs independent of the metastatic organ. In addition, clonal evolution during tumor progression can generate molecular differences between primary tumors and metastatic lesions, leading to variable immune phenotypes across different metastatic sites within the same patient.

Third, the coexistence of metastasis in multiple organs complicates the interpretation of organ-specific immune responses. In many patients with metastatic CRC, liver metastasis occurs alongside lung, lymph node, or peritoneal metastasis, making it difficult to determine whether systemic immune suppression is driven primarily by hepatic immune tolerance or by interactions between multiple metastatic niches.

Finally, much of the mechanistic understanding of PMN formation is derived from experimental models. Although these studies have provided important insights into the cellular and molecular mechanisms underlying metastatic colonization, translating these findings to human disease remains challenging due to species differences, tumor heterogeneity, and the complexity of human immune responses. Further studies integrating spatial transcriptomics, single-cell sequencing, and longitudinal clinical data are therefore required to better characterize organ-specific immune landscapes in metastatic CRC.

## 8 Future directions

Understanding the biological mechanisms underlying organ-specific PMNs may provide new opportunities for improving treatment outcomes in metastatic CRC. One potential strategy involves targeting the molecular signals responsible for PMN formation. Tumor-derived extracellular vesicles, integrin signaling pathways, and cytokine networks that regulate immune cell recruitment represent potential therapeutic targets that could disrupt niche formation before metastatic colonization occurs (22). Future PMN-directed strategies may be implemented clinically by combining biomarkers of metastatic organotropism, including tumor-derived extracellular vesicle profiles, cytokine/chemokine signatures, radiopathomic models, and multi-omic characterization of the primary tumor and circulating tumor products, to predict whether a patient is more likely to develop liver- or lung-dominant disease (104, 105). This distinction is therapeutically relevant because liver metastases are associated with a more tolerogenic, stromal- and myeloid-dominant TME and poorer ICI response, whereas lung-only disease appears more responsive to ICI-based combinations, particularly in MSS mCRC. Therefore, future trials should test site-adapted approaches, such as macrophage/CAF/TGF-β/VEGF-directed strategies for liver-dominant disease and neutrophil-, CXCR2-, NET-, or interferon/STING-modulating strategies for lung-dominant disease, while stratifying outcomes by metastatic site (22, 106).

Another promising approach focuses on reprogramming the immune microenvironment of metastatic organs. In the liver, strategies aimed at reducing immunosuppressive myeloid populations or modulating macrophage polarization may help restore antitumor immunity. Studies have shown that the liver microenvironment can promote systemic immune suppression through interactions between macrophages, regulatory T-cells, and cytotoxic T-cells, suggesting that therapies targeting these pathways may enhance the efficacy of immune checkpoint blockade (14, 53, 55).

Combination treatment strategies may also overcome organ-specific resistance to immunotherapy. Integrating ICIs with radiotherapy, anti-angiogenic therapy, or targeted agents has shown potential to modulate the tumor microenvironment and enhance antitumor immune responses. For example, radiotherapy can induce immunogenic tumor cell death and stimulate systemic immune activation, potentially improving responses to checkpoint blockade in metastatic disease (107). However, so far, studies targeting components of the TME have yielded disappointing results. This highlights the complexity of tumor–immune interactions and suggests that targeting a single component is not sufficient.

Future clinical trials investigating ICIs should prioritize stratifying outcomes based on the presence or absence of specific metastatic sites and, importantly, evaluating responses at the level of individual organs rather than relying solely on overall response rates. Such an approach would provide more precise insight into site-specific therapeutic sensitivity and resistance, particularly given the growing evidence that different metastatic niches exhibit distinct immunologic and microenvironmental profiles. Incorporating organ-specific response assessments into trial design could improve patient selection, guide tailored therapeutic strategies, and ultimately enhance the development of more effective, site-adapted treatment approaches in metastatic colorectal cancer.

## 9 Conclusion

Metastasis remains the leading cause of mortality in CRC, and the biological mechanisms that govern metastatic colonization are increasingly recognized as key determinants of therapeutic response. The formation of the PMN represents a critical early step in this process, enabling primary tumors to modify distant organs and the future TME through systemic signaling, immune modulation, and stromal remodeling before the arrival of circulating tumor cells.

In CRC, the liver and lungs represent the most common sites of metastatic spread, yet they exhibit markedly different immune landscapes. The liver is characterized by a highly tolerogenic microenvironment enriched with immunosuppressive immune cells and regulatory pathways that can dampen antitumor immunity, whereas the lung microenvironment generally supports stronger immune surveillance and inflammatory responses. These organ-specific differences likely contribute to the heterogeneous responses to ICIs observed in metastatic CRC.

Although substantial progress has been made in understanding the cellular and molecular mechanisms underlying organ-specific metastatic niches, many questions remain regarding how these environments evolve during disease progression and treatment. Continued integration of mechanistic studies, advanced molecular profiling technologies, and clinical data will be essential to fully elucidate how PMN biology influences immunotherapy response. Ultimately, a deeper understanding of organ-specific tumor microenvironments may enable the development of therapeutic strategies that overcome organ-dependent resistance and improve outcomes for patients with metastatic CRC.
